# Decoding Medial Entorhinal Cortical Dynamics Produces Planning‐Like Alternations in Hippocampal theta Sequences

**DOI:** 10.1002/hipo.70131

**Published:** 2026-09-18

**Authors:** Masahiro Nakano, Caswell Barry, Claudia Clopath

**Affiliations:** ^1^ Sainsbury Wellcome Centre for Neural Circuits and Behaviour University College London London UK; ^2^ Department of Cell and Developmental Biology University College London London UK; ^3^ Department of Bioengineering Imperial College London London UK

**Keywords:** Bayesian decoding, Hippocampus, medial entorhinal cortex, planning, theta sequences, theta skipping

## Abstract

The hippocampus is central to memory and spatial planning. A prominent candidate mechanism supporting navigational decision making is hippocampal theta sequences—brief (~120 ms) place‐cell sequences representing future trajectories—which have been interpreted as planning signals based on their alternation between maze arms in T‐maze tasks. However, recent work suggests that intrinsic left–right alternation in medial entorhinal cortex (MEC) theta sequences may underlie this phenomenon. Here, we test this hypothesis using a model of MEC theta dynamics in a T‐maze and show that standard Bayesian decoding yields hippocampal theta sequences that alternate between arms. We then analyzed existing simultaneous MEC‐hippocampal recordings from Vollan et al. (2025). We found tight coupling between MEC and hippocampus, along with coordinated left–right alternation, while the animal was performing an alternation task. Notably, this conjoint alternation occurs not only at choice points but also during inbound and forced‐turn trials. These findings suggest that left–right alternation in hippocampal theta sequences is a ubiquitous phenomenon driven by MEC dynamics, challenging the interpretation that these specifically reflect intentional decision‐making or planning processes only.

## 1 Introduction

The hippocampus is essential for both remembering past experiences and using that information to guide future behavior. Damage to this structure causes profound memory deficits in humans and animal models (Scoville and Milner 1957; Olton et al. 1978, 1979), but its role extends beyond memory storage and retrieval. The hippocampus also enables imagination of future scenarios (Schacter et al. 2012), something that is impaired in patients with hippocampal amnesia (Hassabis et al. 2007). This capacity for prospective thinking underlies planning, and planning‐related representations have been identified in the human hippocampus (Addis et al. 2007; Brown et al. 2016).

In rodents, the hippocampus exhibits a prominent 7–12 Hz theta rhythm that emerges from the combined activity of local interneurons and medial septal inputs (Petsche et al. 1962; Hangya et al. 2009; Vandecasteele et al. 2014). Individual hippocampal neurons fire preferentially near the trough of theta cycles (Dragoi and Buzsáki 2006). Place cells—neurons that fire when an animal occupies specific spatial locations—show systematic phase relationships with theta oscillations (O'Keefe and Recce 1993; Skaggs et al. 1996; Burgess et al. 2007). When an animal traverses a place field, the corresponding place cell initially fires at late theta phases, then progressively earlier—a phenomenon known as phase precession (O'Keefe and Recce 1993; Skaggs et al. 1996). At the population level, phase precession corresponds to rapidly progressing spatial trajectories within each theta cycle: nearby locations are represented early in the cycle while progressively distant locations are represented later. These “theta sequences” reflect temporally organized firing of place cell assemblies that sweep ahead of the animal's current position (Dragoi and Buzsáki 2006; Foster and Wilson 2007; Gupta et al. 2012). When Bayesian decoding is applied to these population patterns, the decoded position sweeps forward within each theta cycle, extending well beyond the animal's actual location (Gupta et al. 2012; Feng et al. 2015; Liu et al. 2023). It is widely held that theta sequences temporally compress spatial information into windows suitable for spike‐timing‐dependent‐plasticity (STDP) (Skaggs et al. 1996; Harris et al. 2002, 2003), potentially supporting memory formation. Indeed, disrupting theta sequences impairs memory encoding (Robbe and Buzsáki 2009; Wang et al. 2015; Bolding et al. 2020; Liu et al. 2023).

Theta sequences may also contribute to memory recall and planning (Winson 1978; Arnolds et al. 1980; Kahana et al. 1999; Buzsáki and Moser 2013). Computational models have proposed a framework in which head direction cells, grid cells, place cells, and prefrontal neurons interact to generate look‐ahead probes to support planning (Erdem and Hasselmo 2012, 2014). Since the initial report by Johnson and Redish (2007), various experimental studies have described theta‐related representations that may reflect planning processes in rodents (Johnson and Redish 2007; Wikenheiser and Redish 2015; Kay et al. 2020; Tang et al. 2021, 2026; Comrie et al. 2024; Yu et al. 2026). In particular, Kay et al. (2020) reported theta sweeps extending toward both the left and right arms as animals approached the decision point in a T‐maze alternation task. Although these sweeps did not predict the eventual choice, they may represent the animal evaluating alternative options—a potential neural correlate of planning (Mattar and Lengyel 2022; Kurth‐Nelson et al. 2023). This finding aligns with the idea that theta oscillations may alternate between encoding and recall (Hasselmo et al. 2002), representing the current and the future, or the real and the imagined (Comrie et al. 2022).

The medial entorhinal cortex (MEC) is a major source of input to the hippocampus and is thought to play a key role in temporal coding within the hippocampal network (Suh et al. 2011; Schlesiger et al. 2015; Lasztóczi and Klausberger 2016; Fernández‐Ruiz et al. 2017; Robinson et al. 2017; Davoudi and Foster 2019). A variety of spatially tuned cell types have been identified in MEC, including grid cells, border cells, and object–vector cells (Hafting et al. 2005; Solstad et al. 2008; Høydal et al. 2019; Gardner et al. 2022). MEC activity is also strongly modulated by theta‐band oscillations (Deshmukh et al. 2010; Vollan et al. 2025), and grid cells exhibit phase precession (Burgess et al. 2007). Recent work has demonstrated that MEC itself exhibits theta sequences (Vollan et al. 2025, 2026). During foraging and exploration, these theta sweeps progress forward but with a directional offset: MEC activity alternates between leftward and rightward sweeps across consecutive theta cycles (Vollan et al. 2025). This phenomenon has also been observed during REM sleep, suggesting that it may arise from a circuit‐level mechanism. Equally, theta sweeps have been reported to be modulated by behaviorally salient objects and movement direction (Vollan et al. 2026), suggesting that theta sequences may operate in multiple modes. In both cases, hippocampal representations are broadly aligned with MEC representations in sweep direction and emerge with a temporal delay, raising the possibility that hippocampal theta sweeps are inherited from MEC theta sweeps.

This close temporal coupling makes it important to consider MEC representations when interpreting hippocampal theta sweeps. In the context of an alternation task, Vollan et al. (2025) analyzed MEC theta sweeps while animals performed an alternation task in an M‐shaped maze. They found that MEC theta sweep direction alternated between leftward and rightward across the maze, including the central stem, decision point, and corners. These observations raise the question of whether hippocampal left–right alternating theta sweeps observed in T‐maze tasks reflect active planning within the hippocampus, or whether they simply mirror underlying MEC dynamics (as discussed in Vollan et al. (2025)). However, although hippocampal recordings from the M‐maze task have been made publicly available, simultaneous MEC–hippocampal recordings during this alternation task have not been analyzed, leaving this question unresolved.

Thus, we tested this idea by combining modeling and data analysis. Specifically, we simulated straight left–right alternating MEC sweeps that disregarded the physical T‐shape and sampled spikes from place cells with fields on or near the T‐maze. In this way, by applying Bayesian decoding to the population activity, we obtained hippocampal theta sweeps that appeared to traverse the left and right arms. Consistent with the left–right alternation in MEC, hippocampal sweeps also alternated between the two arms. We then turned to data analysis. Using a previously published dataset (Vollan et al. 2024) associated with the study by Vollan et al. (2025), we found that hippocampal sweeps at the decision point alternated in coordination with MEC. Notably, this left–right alternation also occurred during inbound trials, where the correct choice was fixed, and even at the corner where the animal could only proceed in one direction. Hippocampal left–right alternation was always preceded by MEC representation. These findings support the view that left–right alternation of hippocampal theta sweeps is a ubiquitous phenomenon. We finally discuss experimental approaches to test more directly whether hippocampal theta sweeps constitute neural correlates of planning.

## 2 Materials and Methods

### 2.1 Simulation of Left–Right Sweeps

To simulate left–right alternating theta sweeps and sample place cell and grid cell spikes, we used RatInABox (George et al. 2024). In a T‐maze, we simulated the agent's trajectory using a predefined behavioral pattern: starting at the bottom of the center arm and then running alternately toward the left and right arms. We then modeled the MEC latent trajectory as a second trajectory derived from the simulated agent's behavior. The instantaneous theta phase was computed as a function of time and the simulated theta frequency. During the initial fraction of each theta cycle (parameter α), the MEC latent position matched the agent's position. During the remaining fraction $\left(1-\alpha \right)$, the MEC latent advanced away from the agent's position at a constant speed of $v_{\mathrm{sub}}$. The movement direction was offset by $20^{{}^{\circ}}$ relative to the agent's instantaneous head direction, consistent with experimental observations reported in (Vollan et al. 2025).

Place‐cell populations were defined under two configurations. In the first configuration, place cells were distributed uniformly along the linearized T‐maze trajectory at a constant spatial density (see Table 1). In the second configuration, a two‐dimensional square environment was defined by embedding the T‐maze within a surrounding padding region. Place‐cell centers were positioned on a regular mesh grid at the specified areal density (Table 1). The place cell's instantaneous firing rate was calculated based on the distance between the assigned place field center and the latent position (See Figure S1 for the distribution of place field centers). The firing rate r was calculated as

(1) $$r=r_{\max}\exp\left(-\frac{d^{2}}{2\sigma^{2}}\right)$$

where $r_{\max}$ is the peak firing rate, d is the distance between the place field center and the MEC latent, and σ is the place field width.

**TABLE 1:** Parameters used in the simulation.

	Parameter names		Values	Units
Behavior	Running speed		0.3	ms^−1^
Maze		Center arm length	1	m
		Left and right arm length	0.5	m
		Arm width	0.1	m
General	Time step size (∆t)		1	ms
	Simulation length		20	s
MEC theta sweeps	Theta sequence forward sweep fraction (α)		0.5	
	Theta sequence speed ($v_{\mathrm{sub}}$)		8	ms^−1^
	Theta frequency		10	Hz
Place cells	Density of place cells for simulation 1		500	m^−1^
	Density of place cells for simulation 2		2500	m^−2^
	Place field width (σ)		0.15	m
	Peak firing rate ($r_{\max}$)		20	Hz
Grid cells (multi‐module simulation)	Number of grid modules used		3	
	Grid spacing of each grid module		0.4, 0.56, 0.8	m
	Theta sequence speed of each grid module		4, 5.6, 8	ms^−1^
	Number of grid cells used in each grid module		624, 318, 156	
	Peak firing rate ($r_{\max}$)		40	Hz

Then, binary spikes were sampled with a probability of ρ

(2) ρ=∆t·r

where ∆t is the time step size.

In a second model, we tested whether the left–right theta sweep effect was preserved when the MEC input was modeled as a multi‐module grid‐cell population. We simulated three grid‐cell modules with different spatial periods. Each module was driven by a separate MEC latent whose theta sweep followed the same left–right alternating rule as in the main model, but with sweep distance scaled by the module spacing, such that larger‐scale modules swept over a larger spatial range within each theta cycle. For each grid cell, the firing rate was computed by evaluating a periodic grid tuning function at the module‐specific latent position. The tuning function was defined by the cell's grid scale, orientation, and phase offset, and implemented as a rectified average of three cosine waves separated by 60°. The resulting activity was then scaled between the minimum and maximum firing rates. The number of grid cells in each module was scaled inversely with the square of the module spacing, so that modules with smaller grid spacing contained more cells and modules with larger spacing contained fewer cells. Hippocampal activity was generated as a nonlinear readout of the grid‐cell input. Grid‐to‐place connection was determined by first assigning each hippocampal cell a pseudo‐place‐field center on the T‐maze uniformly, similar to the first model. For each cell, we estimated the average grid‐cell activity near this center and selected the 10 most active grid cells from each module, resulting in 30 grid‐cell inputs per hippocampal neuron. For each of these 30 connected grid cells, the average activity near the pseudo‐place‐field center was multiplied by a random scaling factor drawn from a log‐normal distribution with underlying normal mean 0 and standard deviation 0.25. The resulting activities were then normalized to sum to one and used as the synaptic weight. The weights between the unconnected grid cells and the hippocampal neuron were set to zero. The input drive to hippocampal cell j was calculated as a weighted sum of the selected grid‐cell firing rates,

(3) $$I_{j}\left(t\right)=\sum_{i}W_{\mathrm{ji}}r_{i}^{G}\left(t\right)$$

where $r_{i}^{G}\left(t\right)$ is the firing rate of grid cell i, and $W_{\mathrm{ji}}$ is the feedforward weight from grid cell *i* to hippocampal cell *j*. Hippocampal firing rates were obtained by applying a rectifying nonlinear threshold to this drive,

(4) $$r_{j}^{H}\left(t\right)=r_{\max}{\left[\frac{I_{j}\left(t\right)-\theta_{j}}{s_{j}}\right]}_{+}^{2}$$

where threshold $\theta_{j}$ was chosen to produce sparse activity, that was sampled independently for each neuron from a uniform distribution in range (0.05, 0.15). $s_{j}$ normalized the suprathreshold drive to the maximum firing rate. Spikes were then sampled as in Equation (2).

In the third simulation, we assumed the latent that drives hippocampal activity stays within the maze track, while alternating left and right. Here, when the agent is running toward the decision point, the latent sweeps ahead first, and then turns either left or right and keeps sweeping in that arm. The direction of turn switched every theta cycle. When the agent was already in either left or right arm, the agent swept forward within that arm. Apart from the difference in the latent dynamics, the procedure of simulating hippocampal activity is the same as the main model.

See Table 1 for parameters used in the simulation.

### 2.2 Path Linearization

To linearize positions, six anchor points were defined corresponding to the center bottom, center top (junction), top left, bottom left, top right, and bottom right of the maze. Path points were then interpolated between these anchors, and all behavioral positions were projected onto the nearest path points.

### 2.3 Temporal Binning

In the dataset from Vollan et al. (2025), behavioral measures and neural activity were sampled in 10‐ms bins. To match this, we resampled the simulated dataset in 10‐ms bins.

All analyses were performed on this 10‐ms binned data.

### 2.4 Firing Rate Map/Head‐Direction Tuning Curve

Place fields were estimated in two coordinate spaces. On the linearized path, firing rates were binned at 2 cm resolution and smoothed with a 5 cm Gaussian kernel. Smoothing was constrained within each arm and did not cross boundaries. Linearized fields were used for population decoding in both simulations and data analyses. In two dimensions, the maze was divided into 2 cm × 2 cm bins, and kernel density estimation was applied with a 5 cm bandwidth. These two‐dimensional fields were used for visualization and place‐field correlation analyses (Figures 2, 3, 4).

Circular kernel density estimation was performed using 6° bins and smoothed with a von Mises kernel (κ=10).

In all cases, spike density and position density were calculated separately, and tuning curves were calculated by dividing spike density by position density.

### 2.5 Population Decoding

Population activity was decoded using a Bayesian approach with a uniform prior. Only frames with more than one spike were used in the analysis.

To quantify left–right alternation in the decoded trajectory, we identified time points when the decoded position lay in the left or right arm, respectively. These time series were treated analogously to spike trains. Following the procedure for theta skipping, we computed a skipping index for the left and right latent event trains (see *Theta‐skipping index*).

Theta‐cycle indices were shuffled 1000 times to generate a null distribution. The 5th percentile of this distribution defined the chance level of theta skipping; observed values below this threshold were considered to exhibit left–right alternation.

### 2.6 Theta‐Skipping Index

We adapted the method used in Brandon et al. (2013). For correlation analyses, spikes within each 10 ms bin were binarized. For each spike auto‐ or cross‐correlogram, two peak values were extracted to quantify firing in alternating theta cycles:

(5) $$p_{1}=C\left(60-180\;\mathrm{ms}\right),p_{2}=C\left(180-300\;\mathrm{ms}\right)$$

where $C\left(t_{1}-t_{2}\right)$ denotes the mean spike count within the specified time window $\left[t_{1},t_{2}\right]$.

$p_{1}$ is the mean count of the auto‐ or cross‐correlogram during the time window that corresponds to the next theta cycle, and $p_{2}$ is for the second next theta cycle.

The theta‐skipping Index (TSI) was computed as

(6) $$\mathrm{TSI}=\frac{p_{2}-p_{1}}{\max\left(p_{1},p_{2}\right)}$$

A value of 1 indicates complete theta skipping, whereas −1 indicates firing one theta cycle apart, reflecting theta cycling.

### 2.7 Theta‐Skipping Cells

To generate shuffled controls, spike times were randomized while preserving theta‐phase structure. Theta cycles were first segmented, and their indices were then shuffled so that theta modulation was maintained after shuffling.

Cells were classified as “loose” theta‐skipping if they met two criteria. First, mean firing rates exceeded a minimum threshold (hippocampus: 1 Hz; MEC: 5 Hz). Second, the observed TSI exceeded the 95th percentile of a null distribution generated from 1000 shuffles.

For a stricter classification, cells were additionally required to have TSI>0.5, excluding weak alternators (Figure S2D).

### 2.8 Clustering of Theta‐Skipping Cells

An affinity matrix was constructed using pairwise theta‐skipping indices. Spectral clustering was applied with cluster numbers ranging from 2 to 8, and silhouette scores were computed. The cluster number yielding the highest score was selected.

### 2.9 Firing‐Rate Map and Head‐Direction Tuning Correlation

To assess whether hippocampal firing‐rate maps and MEC head‐direction tuning curves were more similar within clusters than across clusters, we computed pairwise correlation matrices for all cells. The difference between the mean within‐cluster and across‐cluster correlation coefficients defined an index of within‐cluster similarity. This index was recalculated 5000 times with shuffled cluster labels to obtain a null distribution, from which *p* values were derived.

### 2.10 Time‐Lag Analysis

For each hippocampal–MEC cell pair, cross‐correlations were computed within a ±250 ms window. The lag corresponding to the peak correlation was taken as the pair's time lag. Pooling these values across cell pairs yielded the lag distributions plotted in the figures. To statistically assess whether the lag distribution was biased toward MEC leading, we performed a permutation test. We used the proportion of cell pairs with a negative time lag, indicating MEC‐leading activity, as the test statistic. Hippocampal and MEC spike times were independently shuffled, after which the lag distribution and test statistic were recalculated. This procedure was repeated 1000 times to construct a null distribution, from which *p* values were derived.

### 2.11 Comparison of Two Theta Sweep Models

We compared two models of theta sweeps by examining where hippocampal activity peaked along the central stem. In this analysis, we defined place cells with assigned place‐field centers on either the left arm (< −0.3 m) or the right arm (> 0.3 m) as top‐corner cells. We calculated firing‐rate curves during the late theta phase while the agent was running outbound along the central stem, computed the population‐averaged firing rate in each position bin, and identified the peak bin. We then compared the cell‐wise firing rate in this peak bin with the cell‐wise firing rate at the end of the central stem, corresponding to the decision point in the maze. A one‐sided paired *t*‐test was used to ask whether the cell‐wise firing rate in the peak bin was significantly higher than that at the decision point. This comparison was repeated for all position bins, and black dots indicate bins in which the cell‐wise firing rate was significantly higher than at the decision point (Figure S3). The same analysis was then applied to the hippocampal data.

### 2.12 Statistical Analysis

Data analyses were performed with custom‐written scripts in Python, and clustering analyses and statistical analyses were conducted using the python package scipy and scikit‐learn.

## 3 Results

### 3.1 Modeling Left–Right MEC Sweeps Reveals Planning‐Like Alternation in Hippocampal Activity

**FIGURE 1:**
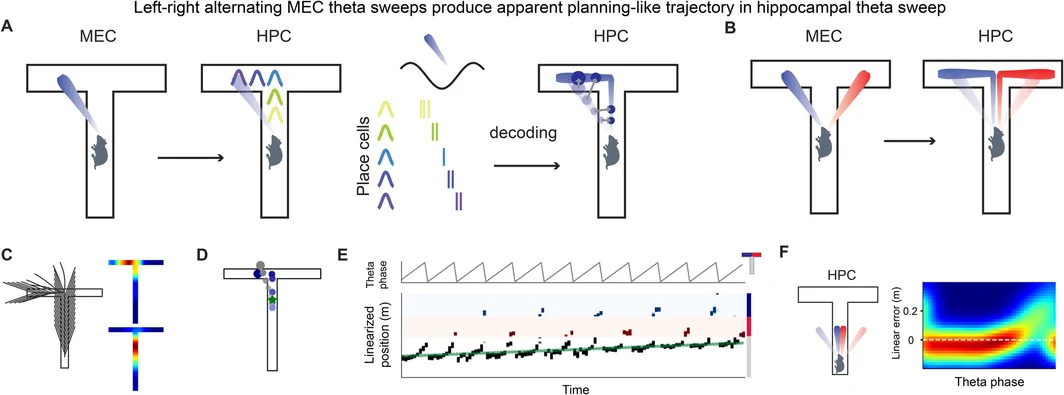
*Modeling left–right MEC sweeps reveals planning‐like alternation in hippocampal activity*. (A, B) Model schematics showing hippocampal (HPC) sequences as projections of MEC sequences onto the T‐maze. (C) Left: Alternating latent sequences used in simulation. Right: Two example hippocampal place cells. (D) Example theta sequence decoding showing current position (green), MEC latent (gray), and decoded position (blue), with opacity indicating temporal progression. (E) Decoded hippocampal sequences alternate between left (blue) and right (red) arms. Top: Theta phase. Bottom: Linearized likelihood map with darker colors showing higher likelihood; colors distinguish center, left, and right arms; green shows actual position. Inset shows the color for each arm. (F) Relationship between theta phase and linear error at the bottom of the center arm. Rat illustration adapted from Sana (2026), *Rat Outline*, SciDraw, CC BY 4.0 (DOI: 10.5281/zenodo.7842073).

We set out to investigate whether left–right sweeps originating from MEC are sufficient to generate hippocampal representations that resemble alternating sweeps along the T‐maze arms (Figure 1A,B). To do so, we built a simple computational model consisting of an agent running down a T‐maze, with the “MEC latent” sweeping left and right (Figure 1C, left). During the early phase of theta, the MEC latent remained local, matching the agent's actual position—during the late phase, it swept ahead of the agent's position by an offset of 20 degrees from the head direction. The offset direction alternated between left and right in consecutive theta cycles (see Materials and Methods). This assumption is based on the findings of Vollan et al. (2025), in which MEC theta sweeps alternated between leftward and rightward directions during the alternation task. This MEC latent drove activity in hippocampal place cells (Figure 1A). Each hippocampal place cell was assigned a place field center uniformly distributed along the linearized track on the T‐maze (Figure S1A; left), and its instantaneous firing rate was determined by the Euclidean distance between the place field center and the MEC latent. Importantly, physical maze boundaries were ignored, so firing rate depended solely on this distance (see example firing rate map in Figure 1C, right—note that the firing rate maps were calculated relative to the agent's position, not the MEC latent).

We next applied Bayesian decoding to infer latent position from place cell activity, using a maximum a posteriori (MAP) decoder. Because the firing rate map was only defined for locations the agent had visited, and decoding assigns the most likely position bin given the population activity, the decoder could not recover the true MEC latent, which sometimes extended beyond the T‐maze (Figure 1A). Instead, the decoder chose the best estimate within the area of the track. Consequently, decoding full MEC latent sweeps directed left while the agent was running in the center arm produced apparent sweeps that extended along the stem and into the left arm (Figure 1D). Hippocampal theta sweeps decoded in the T‐maze near the decision point showed significant alternation between left and right arms, reflecting the alternation in MEC latent (Shuffle test. See Materials and Methods for detail, Figure 1E). We also examined theta sweeps when the agent was far from the decision point, effectively making the track linear. Consistent with the idea of forward left and forward right sweeps projecting onto the linear track, we observed forward‐directed theta sequences (Figure 1F). We then repeated the simulations under the assumption that some place cells had fields outside the physical T‐maze. Place‐field centers were uniformly distributed within the square environment that contained the T‐maze. The square environment was designed so that all latent positions were contained within its boundaries (Figure S1A; right). In this case, too, left–right MEC sweeps produced hippocampal theta sweeps alternating between the two arms (Figure S1A,B). We tested this idea with a second model, which used multiple grid modules projecting to hippocampal neurons (Figure S1C; see Materials and Methods). These grid modules had different grid spacing, and the theta sweeps' length was proportional to their grid spacing. The modules also had coherent sweep directions, based on the findings of Vollan et al. (2025). We used three modules in the simulation. Each hippocampal neuron received input from all three grid modules, and its activity depended on the input it received from the grid cells. Hippocampal neurons showed place‐like activity (Figure S1D). In this simulation, we again found that decoding hippocampal representation produces left–right alternating representations (Figure S1E). Together, these results demonstrate that, under the assumption of MEC theta sweeps driving hippocampal theta sweeps, the combination of Euclidean left–right MEC sweeps and standard Bayesian decoding is sufficient to produce hippocampal theta sweeps that could be interpreted as neural substrates of planning, as originally suggested in Vollan et al. (2025).

### 3.2 Simultaneous MEC–Hippocampal Recordings Reveal Consistent Coupling and Left–Right Alternation at the T‐Maze Decision Point

**FIGURE 2:**
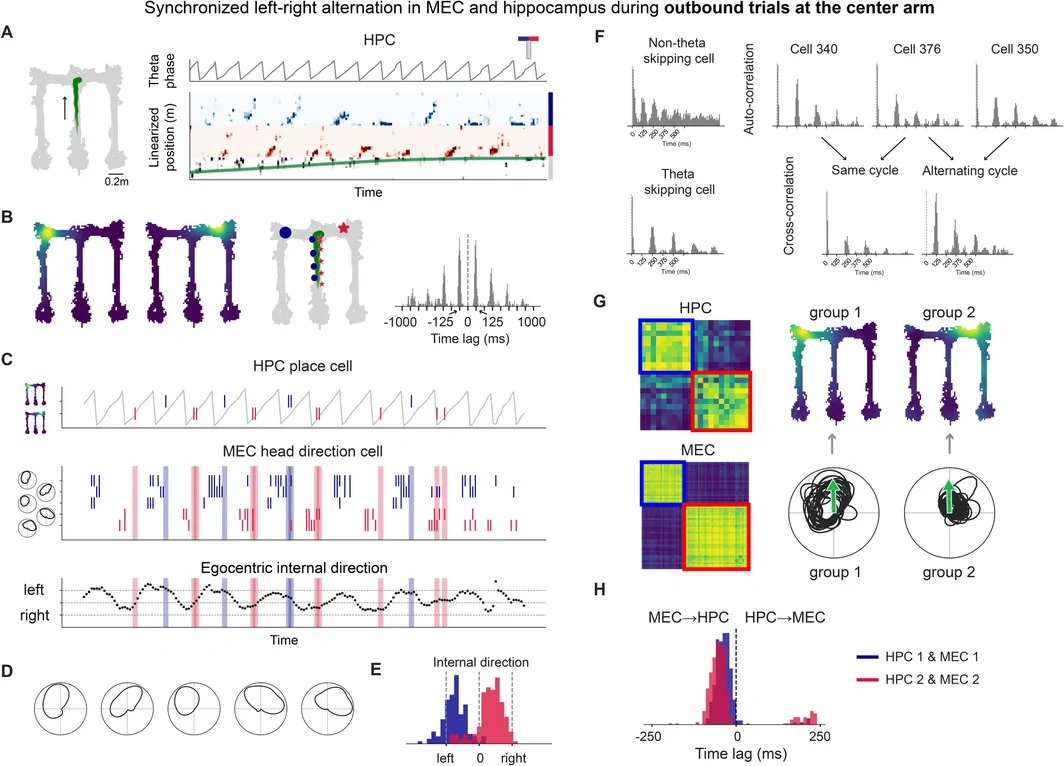
*Simultaneous MEC–hippocampal recordings reveal consistent coupling and left–right alternation at the T‐maze decision point*. (A) Example outbound trial showing the animal's trajectory (left), theta phase (top right), and population decoding likelihood (bottom right). Inset shows the color for each arm. (B) Left: Firing rate maps of two hippocampal cells. Middle: Example outbound trial showing the trajectory (green) and the animal's positions when the two hippocampal cells were active (left; blue, right; red). Large blue circle and red star indicate the place‐field centers. Right: Cross‐correlogram between the two hippocampal cells across all outbound trials. Ticks are shown every 125 ms from −1000 to 1000 ms. (C) Same trial as in (B), showing activity of hippocampal cells (top), MEC cells (middle), and egocentric internal direction—defined as the difference between head direction and MEC internal direction—(bottom). Blue and red shading indicate the spike times of the corresponding hippocampal cells of the top row. (D) Head direction tuning curves of the MEC cells shown in (C). (E) Distribution of internal directions when the two hippocampal cells were active; colors correspond to panel (B). Internal direction of 5 frames before the place cell active is plotted, to reflect the time lag between MEC and hippocampus. (F) Example auto‐correlograms of a non‐theta skipping cell and a theta skipping cell (left), and cross‐correlograms of theta skipping cell pairs active in the same versus alternating theta cycles (right). (G) Theta‐skipping cell analysis showing clustering (left), summed firing‐rate maps for hippocampal cells (top middle), and head‐direction tuning for MEC cells (bottom middle). (H) Distribution of cross‐correlation lags for hippocampus‐MEC cell pairs with matching direction preference.

If hippocampal left–right alternating sequences inherit their structure from MEC, then the timing and direction of sweeps in these regions should match. To test this possibility, we analyzed a publicly available dataset from Vollan et al. (2025) containing simultaneous recordings from MEC and hippocampus while a rat performed an alternation task (Vollan et al. 2024). They used an M‐maze, a variant of the T‐maze in which the left and right arms have an extra turn before the reward well. The dataset included 287 hippocampus cells and 681 MEC and para‐subiculum (PaS) cells from a single animal. PaS exhibits a representation similar to that of MEC but shows stronger modulation by head direction (Boccara et al. 2010; Vollan et al. 2025). Vollan and colleagues reported that grid cells were predominantly found in MEC, whereas head‐direction‐modulated cells were more common in PaS, from the inferred anatomical location of the clusters (Vollan et al. 2025). However, defining grid cells in a maze environment is challenging, partly due to limited spatial sampling and partly due to environmental influences on grid cell firing patterns (Derdikman et al. 2009). Since MEC and PaS directional signals are aligned (Vollan et al. 2025), and our interest lies in the sweeping direction of MEC theta sequences, we included all units recorded in the MEC probe (MEC‐Pas). Using Bayesian acausal population decoding, we first verified that hippocampus place cells exhibited forward sweeping theta sequences along the linear portion of the central arm (Figure S2A). Decoding errors were lowest during the early phase of theta and increased during the late phase of theta, consistent with previous reports on theta sequence in linear tracks (Gupta et al. 2012; Feng et al. 2015; Liu et al. 2023). We then examined activity as the animal approached the decision point (example outbound trial, Figure 2A left). Clear alternation of theta sweeps toward the left and right arms was observed, replicating the original findings (Figure 2A right, shuffle test, *p* < 0.05; 1000 permutations, all outbound trials combined [Kay et al. 2020]). Consistent with this population‐level result, we identified a pair of place cells with place fields in the top‐left and top‐right corners of the maze (Figure 2B left). This pair showed theta‐cycling activity as the animal approached the decision point (Figure 2B middle. Same trial as Figure 2A). Cross‐correlation analysis across all outbound trials confirmed consistent cycling between the two cells (Figure 2B right). Such examples of theta‐paced cross‐correlation between cell pairs have been independently observed in MEC and Pas (Brandon et al. 2013) and in hippocampal cells (Kay et al. 2020). Next, we examined MEC‐PaS representations when hippocampal sweeps alternated between the left and right arms. We found MEC cells that were active immediately before hippocampus place cells (Figure 2C middle). These MEC cells were modulated by head direction, and their preferred direction matched the direction of the place fields of hippocampal cells that followed the activity (Figure 2D).

Vollan and colleagues computed an “internal direction” signal from MEC and PaS activity and showed that, as in open‐field recordings, this internal direction alternated between left and right on the M‐maze (see Figure S8 in Vollan et al. (2025)). We found that, similar to the open‐field results, the left–right alternation of MEC egocentric internal direction, defined as the difference between head direction and MEC internal direction, was consistent with the relative direction of hippocampus place field with respect to head direction (Figure 2C bottom, Figure 2E). Previous reports suggest that hippocampal activity lags behind MEC activity, reflecting the information flow from MEC to hippocampus (Mizuseki et al. 2009; Ólafsdóttir et al. 2016; Vollan et al. 2025). Consistently, place cell activity occurred during the late phase of internal‐direction switching (Figure 2C bottom).

To systematically quantify this phenomenon, we analyzed spike‐time correlations. Many hippocampal and MEC neurons are modulated by the theta rhythm (O'Keefe and Recce 1993; Huxter et al. 2003; Zugaro et al. 2005; Deshmukh et al. 2010), resulting in autocorrelation peaks every 100–120 ms (non‐theta‐skipping cells; Figure 2F top left). In contrast, some cells exhibit peaks separated by two theta cycles (theta‐skipping cells; Figure 2F bottom left). Theta‐skipping cells have been reported in the hippocampus, MEC, thalamus, and PFC (Deshmukh et al. 2010; Brandon et al. 2013; Tang et al. 2021; Vollan et al. 2025) and may contribute to alternating theta sweeps. Notably, theta skipping can occur only in specific spatial contexts. For instance, theta skipping in a given place cell might only be obvious when the animal is running toward the place field center, but this effect is obscured when all time points are included (Figure S2B). We therefore identified theta‐skipping cells within spatially and directionally restricted time windows. First, we restricted the analysis to strongly theta‐skipping cells. Across 44 outbound trials, we detected 21 theta‐skipping hippocampal cells (Figure 2F, top right; see Materials and Methods for details). We then computed cross‐correlations for all identified theta‐skipping cell pairs. Cell pairs with peaks at even theta cycles fired within the same theta cycle, whereas those with peaks at odd theta cycles fired in alternating cycles (Figure 2F, bottom right). From these cross‐correlations, we derived a “theta‐skipping index” for all pairs and constructed a theta‐skipping index matrix (see Materials and Methods). Clustering this matrix revealed two major hippocampal populations (Figure 2G top left, Materials and Methods), corresponding to top‐left and top‐right place cells (Figure 2G top right; Sum of firing rate map of all cells in each group. Individual maps in Figure S2C). Firing rate maps had significantly higher correlation within the group compared to across groups (shuffle test, *p* < 0.05; 5000 permutations, Materials and Methods). Applying the same analysis to MEC theta‐skipping cells again yielded two groups, corresponding to left‐ and right‐direction‐modulated cells (Figure 2G bottom). Similarly, the overlap of the head direction tuning curve was higher within the group compared to across groups (shuffle test, *p* < 0.05). Finally, we measured the peak time lag between MEC and hippocampal groups. Left‐ and right‐direction‐modulated MEC cells consistently preceded the corresponding top‐left and top‐right hippocampal place cells, with an average lag of ~35 ms (Group 1; −38.7 ms, 250 pairs, *p* < 0.05, Group 2; −28.9 ms, 418 pairs, *p* < 0.05, shuffle test, see Materials and Methods, Figure 2H). We repeated this analysis with looser criteria for theta‐skipping cells. In this case, cells that demonstrated weaker but significant theta‐skipping were included (see Materials and Methods and Figure S2D). These cells often showed a local peak in one theta cycle apart, but the peak size was comparable to the second theta cycle peak, which is different from a classical decay over theta cycles in theta‐modulated cells. These contained place cells that had fields in the center arm, but had stronger coupling to either arm place cells. With this criterion too, theta skipping cells in hippocampus and MEC were both clustered into two groups, which corresponded to relative left and right direction, and MEC‐hippocampal coupling was also preserved (shuffle test, hippocampal firing rate map correlation; *p* < 0.05, MEC head direction tuning curve correlation; *p* < 0.05, time lag between MEC and hippocampus; Group 1; −23.4 ms, 900 pairs, *p* < 0.05, Group 2; −17.3 ms, 1292 pairs, *p* < 0.05, shuffle test). In summary, within the same dataset, we show that (1) hippocampal theta sweeps alternate between left and right arms near the decision point, and (2) this alternation is preceded by a matching left–right representation in MEC.

Another way of interpreting the hippocampal left–right alternating theta representation in Figure 2A is that theta sequence sweeps along the maze track (Figure S3B), rather than following the MEC leftward and rightward sweeps (Figure S3A). One possible mechanism for this is firing‐rate adaptation in the hippocampal recurrent circuit (Chu et al. 2024). To ask which model better explains the data, we simulated an alternative model in which hippocampal activity was driven by a latent variable that remains on the maze track and sweeps left and right along the track (Figure S3B; track‐aligned model). We then compared the average late theta‐phase firing rates of place cells in the left and right arms across both models. When the latent is modeled as a fixed leftward and rightward sweeping agent (Figure S3A; forward left–right model), as the animal approaches the decision point, the theta sweep latent would “overshoot,” causing lower activity at the decision point. In contrast, if the latent sweeps along the track, then as the animal approaches the decision point, the latent should reach the top corners more reliably and thus produce stronger activity. Analysis of the simulated activity confirmed this prediction. In the forward left–right model, the average firing rate peaked before the agent reached the decision point (Figure S3A). To quantify this pre‐decision‐point peak, we identified the bin with the highest population‐average firing rate and compared cell‐wise firing rates in this bin with those at the decision point. Firing rates were significantly higher at the pre‐decision point than at the decision point (paired one‐sided *t*‐test, *t*(199) = 4.91, *p* < 10^−6^). In contrast, in the track‐aligned model, the population‐average firing rate continued to increase and reached its maximum at the decision point (Figure S3B). We then turned to real data. By analyzing the average firing rate of 21 theta‐skipping cells during outbound trials (Figure S2C), we found that activity peaked before the animal reached the decision point. Cell‐wise firing rates were significantly higher at this pre‐decision‐point peak than at the decision point (*t*(20) = 1.95, *p* < 0.05). Therefore, the data are better explained by a model in which the latent position driving hippocampal spikes shows alternating leftward and rightward sweeps, rather than sweeping along the maze track.

### 3.3 MEC‐Hippocampal Coupling Occurs Under Less Cognitively Demanding Conditions

**FIGURE 3:**
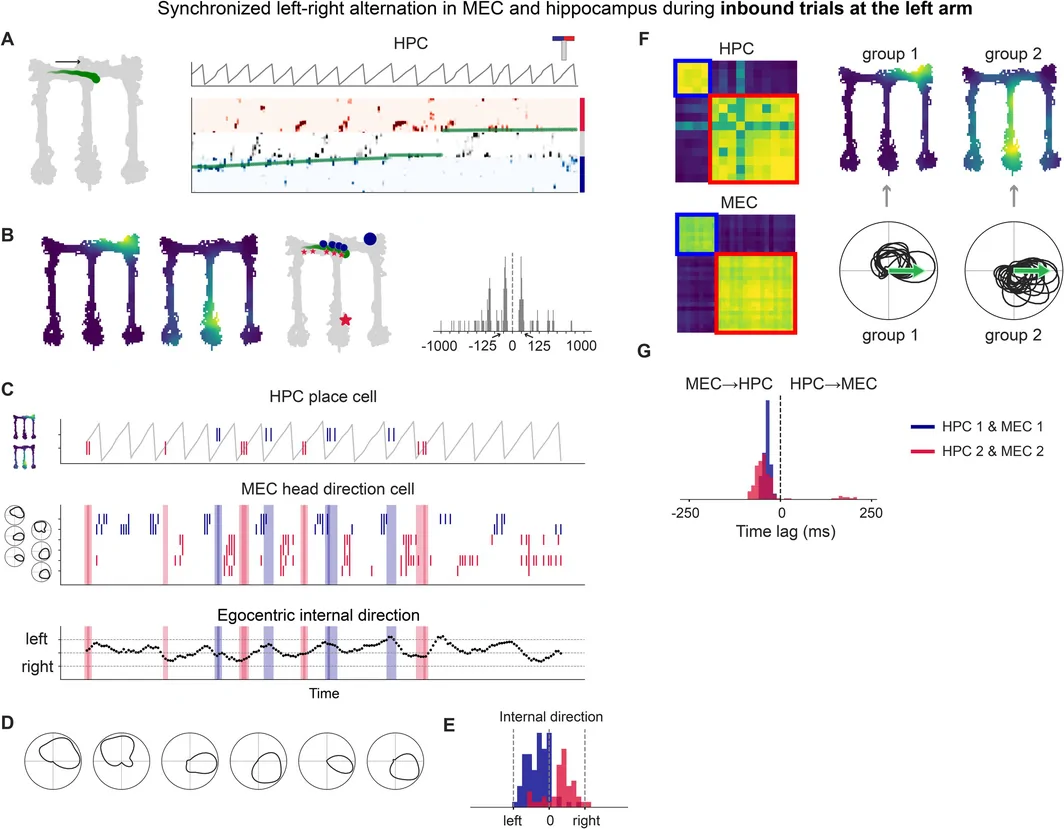
*MEC‐hippocampal coupling occurs under less cognitively demanding conditions*. (A) Example inbound trial when the animal was running toward the junction from the left arm, showing the animal's trajectory (left), theta phase (top right), and population decoding likelihood (bottom right). Inset shows the color for each arm. (B) Left: Firing rate maps of two hippocampal cells. Middle: Example inbound trial from the left arm showing the trajectory (green) and the animal's positions when the two hippocampal cells were active (top‐right; blue, center arm; red). Large blue circle and red star indicate the place‐field centers. Right: Cross‐correlogram between the two hippocampal cells across all inbound trials from the left arm. (C) Same trial as in (B), showing activity of hippocampal cells (top), MEC cells (middle), and egocentric internal direction– defined as the difference between head direction and MEC internal direction– (bottom). Blue and red shading indicate the spike times of the corresponding hippocampal cells of the top row. (D) Head direction tuning curves of the MEC cells shown in (C). (E) Distribution of internal directions when the two hippocampal cells were active; colors correspond to panel (B). Internal direction of five frames before the place cell active is plotted, to reflect the time lag between MEC and hippocampus. (F) Theta‐skipping cell analysis showing clustering (left), summed firing‐rate maps for hippocampal cells (top middle), and head‐direction tuning for MEC cells (bottom middle). (G) Distribution of cross‐correlation lags for hippocampus‐MEC cell pairs with matching direction preference.

In alternation tasks, neural representations have predominantly been studied at the decision point, where cognitive demand is highest and planning is most relevant. However, MEC left–right alternations occur ubiquitously across the M‐maze (Vollan et al. 2025). Building on the idea that hippocampal theta sweeps may reflect MEC sweeps, we asked whether MEC–hippocampal synchronization and left–right alternation extend beyond the decision point. We focused on two additional conditions, beginning with the inbound trials. During inbound trials, when the animal returns from either arm, the task rule requires it to always turn into the center arm—thus demanding less planning or decision‐making. Indeed, animals learn inbound trials more rapidly than outbound trials (Jadhav et al. 2012), and perturbations that impair learning have a smaller effect on inbound trials (Jadhav et al. 2012; Joshi et al. 2026). If hippocampal theta sweeps are closely tied to cognitive demands or planning states, they might be biased toward the correct choice, or show reduced sampling‐like in these low‐demand trials. We examined inbound trials in which the animal returned from the left arm. Similar to the outbound center‐arm trials, we observed alternating theta sequences that swept toward the center and right arms (Figure 3A). Consistent with this, a hippocampal place‐cell pair showed alternating activity (Figure 3B), with place fields centered in the right and center arms, respectively. In the MEC, we identified direction‐selective cells tuned to rightward and downward movements that were active immediately before the corresponding hippocampal place cells (Figure 3C,D). Egocentric internal direction was also consistent with the direction of the hippocampal place fields (Figure 3E). Applying the same theta‐skipping analysis revealed two distinct cell groups in both hippocampus and MEC (Figure 3F left). Each hippocampal and MEC group exhibited correlated place fields and head‐direction tuning curves, respectively (Figure 3F right, shuffle test; hippocampus, *p* < 0.05, MEC, *p* < 0.05). Moreover, MEC groups with egocentric direction selectivity matching the downstream hippocampal place fields consistently preceded hippocampal activity (Figure 3G, shuffle test; Group 1, *p* < 0.05, Group 2, *p* < 0.05). Similar results were obtained using looser criteria for theta skipping cells (tuning curve correlation; hippocampus, *p* < 0.05, MEC, *p* < 0.05, time lag; Group 1, *p* < 0.05, Group 2, *p* < 0.05). Similar results were obtained for inbound trials from the right arm (Figure S4, strict threshold; tuning curve correlation; hippocampus, *p* < 0.05, MEC, *p* < 0.05, time lag; Group 1, *p* < 0.05, Group 2, *p* < 0.05).

### 3.4 MEC‐Hippocampal Coupling Persists Even at Forced Turn Corners

**FIGURE 4:**
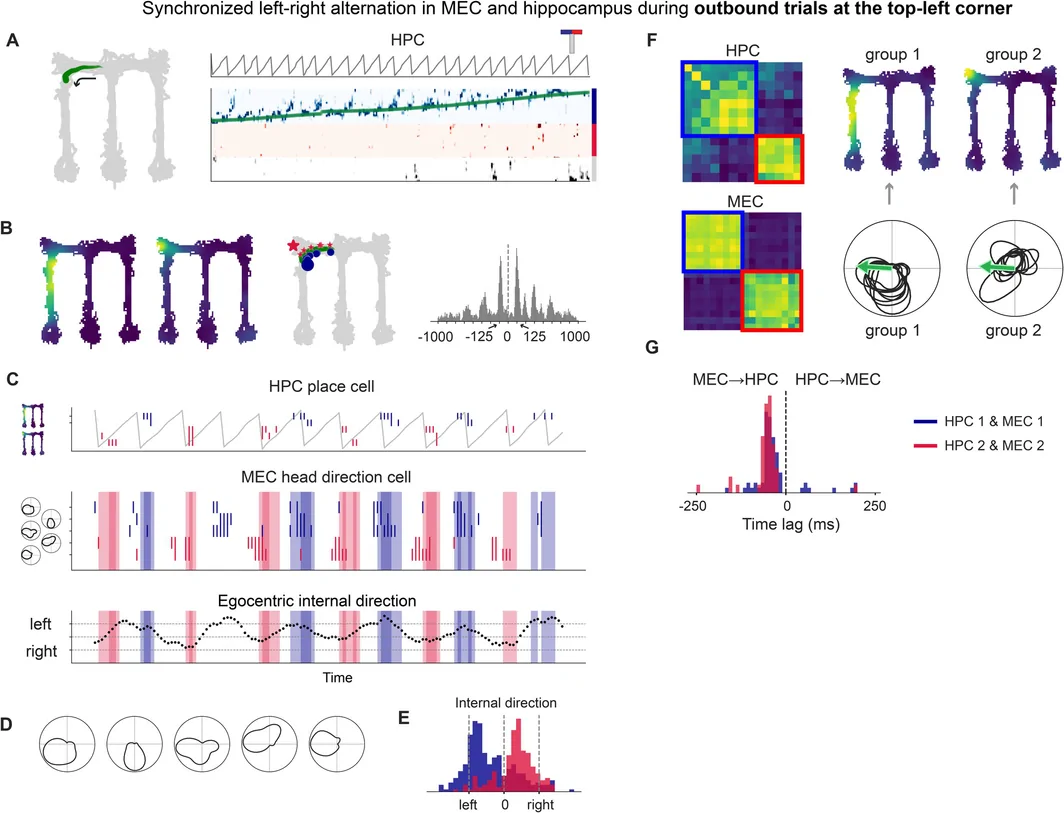
*MEC‐hippocampal coupling persists even at forced turn corners*. (A) Example outbound trial when the animal was running toward the top‐left corner from the junction, showing the animal's trajectory (left), theta phase (top right), and population decoding likelihood (bottom right). Inset shows the color for each arm. (B) Left: Firing rate maps of two hippocampal cells. Middle: Example outbound trial toward the left arm showing the trajectory (green) and the animal's positions when the two hippocampal cells were active (inner corner; blue, top‐left corner; red). Large blue circle and red star indicate the place‐field centers. Right: Cross‐correlogram between the two hippocampal cells across all outbound trials toward the left arm. (C) Same trial as in (B), showing activity of hippocampal cells (top), MEC cells (middle), and egocentric internal direction– defined as the difference between head direction and MEC internal direction—(bottom). Blue and red shading indicate the spike times of the corresponding hippocampal cells of the top row. (D) Head direction tuning curves of the MEC cells shown in (C). (E) Distribution of internal directions when the two hippocampal cells were active; colors correspond to panel (B). Internal direction of five frames before the place cell active is plotted, to reflect the time lag between MEC and hippocampus. (F) Theta‐skipping cell analysis showing clustering (left), summed firing‐rate maps for hippocampal cells (top middle), and head‐direction tuning for MEC cells (bottom middle). (G) Distribution of cross‐correlation lags for hippocampus‐MEC cell pairs with matching direction preference.

To study how ubiquitous MEC and hippocampus coupling is, we took advantage of the M‐shaped design of the maze, which introduced a second turning corner absent in a T‐maze. This corner is typically not considered a decision point, as there is only one possible path. Consequently, cognitive demand should be lowest here compared to either the outbound or inbound decision points. We analyzed neural representations when the animal ran from the center arm toward the top‐left corner (Figure 4A). In most trials (15/20), the animal made a smooth turn at the corner and continued down the left arm, while in a subset of trials (4/20), it briefly visited the top‐left corner before descending (Figure S5A). Linearized population decoding results showed theta sweeps progressing in the forward direction along the left arm (Figure 4A). Surprisingly, we identified a hippocampal place‐cell pair exhibiting theta cycling (Figure 4B). The place fields of these two cells corresponded to the left arm and the top‐left corner, which aligned with the animal's leftward and rightward directions, respectively. We found MEC cells that were selective to bottom left and top left direction, that were active immediately before left arm and top‐left corner place cells, respectively (Figure 4C,D). Egocentric internal direction was consistent with the timing of place cell activity (Figure 4E). Applying the same analysis as before, we again identified two groups in both hippocampus and MEC: one corresponding to the top‐left corner and one to the left arm (Figure 4F). The MEC groups showed leftward and rightward direction selectivity and consistently preceded the activity of the matching hippocampal groups (Group 1, *p* < 0.05, Group 2, *p* < 0.05, Figure 4G). Kay et al. (2020) reported theta‐skipping cell pairs in a similar condition, noting that these pairs consisted of cells with outbound‐ and inbound‐dominant responses. Our results, derived from large‐scale recordings, offer an alternative interpretation that emphasizes the spatial location of place fields rather than directional dominance.

This pattern was replicated across most corners of the maze, including the top‐left inbound, top‐right outbound, and top‐right inbound directions (Figure S5B–D). In the top‐right corner inbound condition, there were fewer theta‐skipping cells that met the criteria, and the overlap of firing‐rate maps of hippocampal cells was non‐significant (shuffle test, *p* = 0.111). However, visual inspection suggested the tendency for hippocampal cell groups following left‐ and right‐direction MEC cell activity to have relatively left‐ and right‐directed place fields, respectively (Figure S5D right). Thus, MEC–hippocampal synchronization and left–right alternation occur ubiquitously throughout the maze environment, extending beyond classical decision points.

## 4 Discussion

Using a computational model, we demonstrated that combining left–right alternating theta sweeps with standard Bayesian decoding can produce an apparently planning‐like representation in the hippocampus. Analysis of an existing dataset collected by Vollan et al. (2025) further revealed that during left–right alternating hippocampal theta sweeps in the M‐maze, MEC representations are directionally aligned with, and critically, precede hippocampal activity—indicating robust MEC–hippocampal coupling. Importantly, this synchronization persists beyond the classic decision point, extending to cognitively less demanding inbound trials—where the correct turn is always predetermined—and even to maze corners, where no choice is required. These findings extend previous reports of MEC–hippocampal coordination in open‐field environments by demonstrating that such synchronization also occurs during structured maze tasks and active behavior. Finally, we observed that even when the environment is designed to be “one‐dimensional,” the rat brain represents it in a two‐dimensional manner (Figure 4). This suggests that the common practice of linearizing trajectories in hippocampal studies may obscure important aspects of spatial coding and directional representation.

Several findings from Vollan et al. (2025) already foreshadowed our conclusion. Specifically, Vollan et al. (2025) showed that MEC theta sweep directions alternate between leftward and rightward throughout the M‐maze and provided evidence for tight coupling between MEC and hippocampal representations in open arena and linear track settings, with MEC activity preceding hippocampal activity. Analyzing simultaneous MEC–hippocampal recordings during an alternation task in the M‐maze, we provide an important bridge between these two findings: MEC and hippocampal representations are strongly coupled during the alternation task, and both exhibit left–right alternating sweep directions.

More recently, Vollan et al. (2026) reported that theta sweeps in MEC and PaS can be modulated by behaviorally salient directions, such as the direction of food bait or the animal's movement direction, revealing a more flexible mode of theta sweeps. This finding is in line with computational models proposed by Erdem and Hasselmo (2012, 2014), in which a system comprising head direction cells and multi‐scale grid cells generates look‐ahead probe trajectories and interacts with hippocampal and prefrontal neurons to guide goal‐directed behavior. Results from Vollan et al. (2026) extend these models by showing that such look‐ahead trajectories are not restricted to the forward direction. It further raises the possibility that, under different experimental conditions, MEC representations during alternation tasks may also exhibit such flexible sweep dynamics. How MEC representations are organized when multiple future choices are available therefore remains an important open question. However, the consistent left–right alternation observed throughout the M‐maze in the data we analyzed can be more consistent with a fixed‐alternation mode.

Recent experiments have adopted environments with greater spatial freedom to study hippocampal theta sequence representations, such as complex mazes (Ormond and O'Keefe 2022; Comrie et al. 2024; Yu et al. 2026) or open‐field arenas (Tang et al. 2026). Comrie et al. (2024) reported theta representations that were flexibly modulated by task rules and rewards that changed within a session, including representations of physically remote locations–features that are difficult to reconcile with the Euclidean framework of MEC representations if the hippocampus is merely inheriting them. Yu et al. (2026) and Tang et al. (2026) similarly reported theta sweep directions biased toward goals, and both studies used attractor network models incorporating an egocentric goal signal. Importantly, both models relied on short‐term firing rate adaptation to generate theta sweeps (Ji et al. 2025; Widloski et al. 2025), consistent with treating the MEC as a fully two‐dimensional Euclidean system. However, it remains unclear how the same system could produce meaningful theta sweeps in the M‐maze, where, during outbound trials in the center arm, the goal location is behind the animal. Overall, theta‐related representations ranging from simple left–right alternation to more complex, task‐modulated patterns have been reported. This suggests that theta activity may encompass multiple forms of representations, some of which could support planning. Most importantly, future experiments addressing this question must consider that seemingly non‐cognitive dynamics in MEC could give rise to planning‐like signals, potentially confounding interpretations, and should be designed accordingly.

**FIGURE 5:**
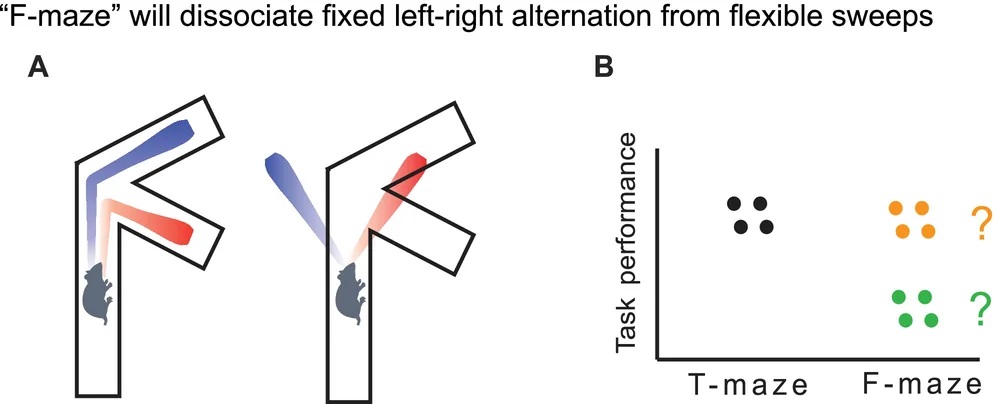
“F‐maze” will dissociate fixed left–right alternation from flexible sweeps. (A) Schematics showing two possibilities of theta sweeps in the F‐maze. Theta sweeps may alternate between two choice arms (left) or alternate between left and right (right). (B) Schematics showing two possibilities of task performance in the F‐maze. Animals may show lower performance, potentially linked to theta sweeps (light green), or show a similar level of performance (orange). Rat illustration adapted from SciDraw.

To disentangle these possibilities, we propose an “F‐maze” (Figure 5). Topologically, it is identical to the T‐ or M‐maze but differs in its physical layout: instead of two choice arms positioned left and right of the center arm, the F‐maze places both arms on one side (in this case, the right). This design would allow us to test two key questions:

1. Do we still observe alternating theta representations across the two choice arms?
2. Does behavioral performance remain comparable to that in the T‐maze, or does it become more difficult for the animals?

If alternating theta representations are observed between the two right‐side arms, this would argue against the idea that hippocampal left–right theta sweeps merely reflect fixed‐mode MEC dynamics (Figure 5A). Such results would provide strong evidence that theta sweeps are modulated by task content during the alternation task, regardless of whether this effect originates in the MEC, hippocampus, or an upstream region. Determining the source of this modulation would require multi‐regional recordings, which could distinguish between changes in MEC dynamics and independent task‐related contributions from the hippocampus. Conversely, if alternating representations are absent in the F‐maze, two interpretations are possible. One is that left–right sweeps are a byproduct of fixed‐mode MEC dynamics, but the brain can nevertheless use this representation for planning in downstream regions. In this case, performance in the T‐maze may be higher because these representations aid planning, whereas performance in the F‐maze would decline due to the loss of supporting information, or at least be different since animals need to use alternative mechanisms. Alternatively, if hippocampal theta sweeps are not used for planning—either within hippocampus or downstream areas—performance should remain comparable between maze types. In this scenario, the brain might instead rely on “splitter cells,” found in multiple regions including hippocampus, prefrontal cortex, entorhinal cortex, and thalamus (Frank et al. 2000; Wood et al. 2000; Ito et al. 2015).

Finally, if theta is not primarily or solely used for memory recall or planning, what is its functional role? Theta's involvement in encoding is increasingly well established, as perturbation studies have demonstrated memory impairments following theta disruption (Quirk et al. 2021; Zhang et al. 2021; Gedankien et al. 2025). Quirk et al. (2021) reported that perturbing the medial septum impaired performance only when applied during the return arm, not during the outbound center arm. Similarly, Zhang et al. (2021) found that medial septum stimulation during the outbound center arm had no effect on performance, whereas stimulation in the delay area reduced ripple rates and task accuracy. These findings suggest that theta oscillations are involved in memory encoding, whereas retrieval may rely on replay mechanisms. Future experiments should therefore employ carefully designed behavioral tasks combined with causal manipulations to determine whether or to which extent theta also contributes to memory recall and planning.

There are several limitations of this study. Most importantly, due to the data availability, the analysis was conducted based on a single animal. Data from additional animals will be required to fully establish the veracity of these findings. Second, we modeled the MEC–hippocampal circuit using a simplified model with many assumptions, including that MEC theta sweeps drive hippocampal theta sweeps. While several studies have indicated this possibility using simultaneous recordings (Mizuseki et al. 2009; Vollan et al. 2025) and lesion studies (Hafting et al. 2008), we still lack definitive evidence. Moreover, there is evidence that the hippocampus is more than a mere follower of MEC, including differing responses to small environmental changes in MEC and the hippocampus (Fyhn et al. 2007). Nonetheless, the fact that this simplified model reproduces key features of the observed hippocampal dynamics suggests that the MEC–hippocampal pathway is sufficient to account for the planning‐like sequences we report, regardless of whether the relationship is strictly feedforward.

In summary, our computational model confirmed the hypothesis that left–right sweeps in the MEC can give rise to planning‐like activity in the hippocampus. Analysis of simultaneous MEC–hippocampal recordings revealed that their coupling occurs ubiquitously during maze alternation behavior, supporting the idea that hippocampal left–right sweeps may arise from MEC‐driven dynamics.

## Funding

This work was supported by the Biotechnology and Biological Sciences Research Council (BB/Y011775/1), the Wellcome Trust (200790/Z/16/Z), the Simons Foundation (564408), the Engineering and Physical Sciences Research Council (EP/R035806/1), and the European Research Council (MotorAdapt 101169605).

## Conflicts of Interest

The authors declare no conflicts of interest.

## Supporting information


**Figure S1:** (A) Distribution of simulated place cells under two assumptions: one in which place cells are confined to locations within the maze, and another in which they also exist outside the maze. (B) Population decoding result for the condition where place cells extended beyond the maze boundaries. (C) Schematic of multi‐scale theta sweeps. All three MEC latent shared the same sweep direction and differed only in sweep length. (D) Example rate map of hippocampal cells receiving inputs from multiple grid modules. (E) Population decoding result of the hippocampal activity when hippocampal cells received inputs from multiple grid modules.
**Figure S2:** (A) Forward theta sweep in the center arm. Left: Animal's trajectory corresponding to the analyzed timepoints. Inset shows the color code for head direction. Right: Decoded population activity during these time points. (B) An example hippocampal cell showing strong theta skipping restricted to a specific region. Left: Firing‐rate map of the cell. Middle: Using all time points. Right: Using time points when the animal was running outbound in the center arm. Same color code as in (A). (C) Individual firing‐rate maps of hippocampal cells belonging to the identified cluster. (D) An example cell that satisfied the loose but not the strict criteria for theta skipping.
**Figure S3:** Comparing two models of theta sweeps: forward left–right sweeps (A) and track‐aligned sweeps (B). (A) Left: Schematic of the forward left–right sweeps. Right: Firing rates of left‐ and right‐arm place cells during outbound trials. The inset shows the corresponding linearized position with different colors. The gray lines show individual‐cell firing rates. The black line shows the population‐average firing rate. Black dots indicate position bins where cell‐wise firing rates were significantly higher than those in the bin closest to the decision point. (B) Same as in (A), but for the track‐aligned sweep model. There was no position bin where cell‐wise firing rates were significantly higher than those in the bin closest to the decision point. (C) Same as in (A), but for real hippocampal data. The inset shows the corresponding linearized position on the center stem.
**Figure S4:** (A) Individual firing‐rate maps of hippocampal cells belonging to the identified cluster. (B) Results for inbound trials when the animal was running from the right arm.
**Figure S5:** (A) Two example trajectories at the maze corner. The animal typically turns smoothly at the corner (left) but occasionally enters the dead end before turning (right). (B) Results for inbound trials from the left arm at the top‐left corner. (C) Results for outbound trials to the right arm at the top‐right corner. (D) Results for inbound trials from the right arm at the top‐right corner.

## Data Availability

Data analyzed in this paper are available at EBRAINS, 10.25493/R5FR‐EDG. Code for reproducing the simulation and analyses in this article will be available at Zenodo 10.5281/zenodo.18340856.
