# Supplementary material for: Decoding Medial Entorhinal Cortical Dynamics Produces Planning‐Like Alternations in Hippocampal theta Sequences
Source: Hippocampus. 2026 Sep 18;36(5):e70131. doi: 10.1002/hipo.70131 (PMC13588937; doi:10.1002/hipo.70131)
Supplement: Supplementary file 1 [file HIPO-36-0-s001.docx]

**Fig. S1.**

(**A**) Distribution of simulated place cells under two assumptions: one in which place cells are confined to locations within the maze, and another in which they also exist outside the maze. (**B**) Population decoding result for the condition where place cells extended beyond the maze boundaries. (C) Schematic of multi-scale theta sweeps. All three MEC latent shared the same sweep direction and differed only in sweep length. (D) Example rate map of hippocampal cells receiving inputs from multiple grid modules. (E) Population decoding result of the hippocampal activity when hippocampal cells received inputs from multiple grid modules.

**Fig. S2.**

(**A**) Forward theta sweep in the center arm. Left: Animal’s trajectory corresponding to the analyzed timepoints. Inset shows the color code for head direction. Right: Decoded population activity during these time points. (**B**) An example hippocampal cell showing strong theta skipping restricted to a specific region. Left: Firing-rate map of the cell. Middle: Using all time points. Right: Using time points when the animal was running outbound in the center arm. Same color code as in (A). (**C**) Individual firing-rate maps of hippocampal cells belonging to the identified cluster. (**D**) An example cell that satisfied the loose but not the strict criteria for theta skipping.

**Fig. S3.**

Comparing two models of theta sweeps: forward left–right sweeps (A) and track-aligned sweeps (B).

(**A**) Left: Schematic of the forward left–right sweeps. Right: Firing rates of left- and right-arm place cells during outbound trials. The inset shows the corresponding linearized position with different colors. The gray lines show individual-cell firing rates. The black line shows the population-average firing rate. Black dots indicate position bins where cell-wise firing rates were significantly higher than those in the bin closest to the decision point. (**B**) Same as in (A), but for the track-aligned sweep model. There was no position bin where cell-wise firing rates were significantly higher than those in the bin closest to the decision point. (**C**) Same as in (A), but for real hippocampal data. The inset shows the corresponding linearized position on the center stem.

**Fig. S4.**

(**A**) Individual firing-rate maps of hippocampal cells belonging to the identified cluster. (**B**) Results for inbound trials when the animal was running from the right arm.

**Fig. S5.**

(**A**) Two example trajectories at the maze corner. The animal typically turns smoothly at the corner (left) but occasionally enters the dead end before turning (right). (**B**) Results for inbound trials from the left arm at the top-left corner. (**C**) Results for outbound trials to the right arm at the top-right corner. (**D**) Results for inbound trials from the right arm at the top-right corner.
